# Characterization and Establishment of an Immortalized Rabbit Melanocyte Cell Line Using the SV40 Large T Antigen

**DOI:** 10.3390/ijms20194874

**Published:** 2019-09-30

**Authors:** Yang Chen, Shuaishuai Hu, Manman Wang, Bohao Zhao, Naisu Yang, Jiali Li, Qiuran Chen, Ming Liu, Juan Zhou, Guolian Bao, Xinsheng Wu

**Affiliations:** 1 College of Animal Science and Technology, Yangzhou University, Yangzhou 225009, Jiangsu, China; yangc@yzu.edu.cn (Y.C.); 18852726848@163.com (S.H.); wmm171717@126.com (M.W.); zhao598841633@163.com (B.Z.); yangnaisu@foxmail.com (N.Y.); li1193117036@163.com (J.L.); m15262241602@163.com (Q.C.); mLiu1994@163.com (M.L.); z76249032@163.com (J.Z.); 2 Joint International Research Laboratory of Agriculture & Agri-Product Safety, Yangzhou University, Yangzhou 225009, Jiangsu, China; 3 Animal Husbandry and Veterinary Research Institute Zhejiang Academy of Agricultural Sciences, Hangzhou 310021, Zhejiang, China; GuolianB@126.com

**Keywords:** melanocytes, immortalization, SV40-LT, cell lines, malignant transformation

## Abstract

Melanocytes (MCs) are specialized cells that synthesize melanin within the melanosome. Cultured MCs are useful in order to study their role in relation to pigmentation. However, MC isolation is laborious and the obtained cells have a limited culture time. In this study, we transformed lentivirus-mediated simian virus 40 Large T (SV40-LT) into primary rabbit melanocytes (Pri RMCs) to establish an immortalized cell line. Morphologically, the immortalized RMCs (Im RMC) were indistinguishable from the Pri RMCs, and dendrites were visible following Dopa staining. No significant differences in cell proliferation or growth between immortalized and primary RMCs were observed. Based on melanocyte-specific markers, the expression of MITF, TYR, and TYRP1 were detected by PCR, immunofluorescence staining, and western blot analysis. Through karyotype, soft agar, and tumorigenesis assays, the immortalized RMCs did not undergo malignant transformation. Our results show that Im RMCs can be used as a tool cell for future MC studies on the pigmentation mechanisms of fur animals.

## 1. Introduction

Epidermal melanocytes (MCs) are specialized melanin-producing cells that synthesize melanin within the melanosome [1,2,3,4]. The coat color in mammals is controlled by melanin distribution and type [5,6]. In the rabbit industry, white coats must be dyed prior to the production of clothing and other products, which impacts the environment and consumer health. Natural colored coats are most popular with consumers. The analysis of rabbit coat pigmentation therefore provides a theoretical basis for the breeding of new coat color varieties. However, to-date, immortalized rabbit melanocytes (Im RMCs) have not been established, limiting our knowledge of melanin deposition in rabbits.

The primary culture and serial passage of MCs in vitro remains limited. In response to tumor suppressor p53 and retinoblastoma suppressor gene (pRB) overexpression, isolated primary cells remain in senescence stages (M1 phase) and their growth is inhibited [7]. MC growth can be stimulated by external factors including viral genes that stimulate M1 progression, leading to immortalization and indefinite cell division in vitro. Exogenous genes commonly used to establish immortalized cell lines include Epstein–Barr virus, human papilloma virus (HPV), simian virus 40 Large T (SV40-LT), and telomerase reverse transcriptase [8].

The simian virus SV40 was discovered in 1960 and belongs to the eukaryotic cell virus, which consists of structural proteins (VP1, VP2, and VP3) and two antigens (large T and small T) [9,10]. The large T antigen of SV40 is often used for genome integration leading to immortalization through cell cycle regulation [11]. The T-antigen binds to a variety of proteins including p53, pRB, DNA polymerase alpha-primase, and heat shock chaperone 70 (hsc70) to prolong the cell cycle and promote cell immortalization [10,12,13]. To-date, an array of immortalized cell lines have been produced using SV40-LT antigen delivery through virus infection, electroporation, liposome-mediated transfection, and viral expression vectors [14,15,16,17].

In previous studies, RMCs were isolated through the collection of rabbit skin tissue and separation by two-step enzymatic digestion [18]. The process was time-consuming and the obtained primary melanocytes could be cultured for a limited time and grew slowly after multiple passages. To solve this issue, we transformed lentivirus-mediated SV40-LT into primary melanocytes and used puromycin to screen to stably integrated SV40-LT cells for unlimited immortalization. The established Im RMCs provide material for in vitro MC experiments and serve as a model for further studies on the molecular mechanisms of pigmentation in fur animals.

## 2. Results

### 2.1. Immortalization of RMCs Using SV40-LT Lentiviral Expression

**Figure 1:**
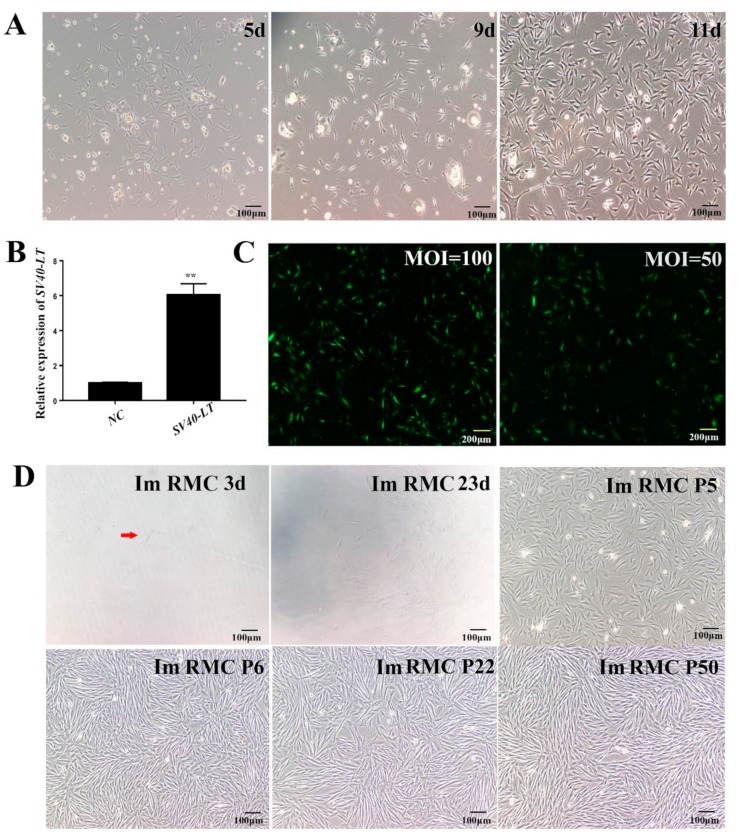
Immortalization of rabbit melanocyte cells using simian virus 40 Large T (SV40-LT) lentiviruses (**A**) Isolation and morphological observations of primary melanocytes (100×). (**B**) Identification of LV-SV40LT lentivirus-expressing cells. PLVX-IRES-Puro was used as normal control (NC, 40×). An extremely significant difference was signified with “**” (p < 0.01). (**C**) Fluorescence analysis of pLVX-IRES-Puro-GFP infection after 48 h. (**D**) Monoclonal selection in which 3d/23 d indicates the 3rd/23th day after monoclonal selection. Im RMC P5/P6/P22/P50 indicates the Im RMCs at passage 5/6/22/50 (100×). Arrows indicate monoclonal cells. MOI: multiplicity of infection; Im RMC: immortalized rabbit melanocytes.

The primary melanocytes of rabbits were isolated by two-step enzymatic digestion. After expansion, keratinocytes and fibroblasts gradually disappeared and pure melanocytes were obtained which were multipolar, bipolar or tripolar with strong refractive indexes (Figure 1A). The pLVX-IRES-Puro-SV40LT lentiviral expression vector was constructed and transfected into 293T cells. Cell supernatants were collected and concentrated with Concen Solution. The lentiviruses were infected into HEK293 cells and gene expression was confirmed by RT-qPCR. The Ct values were ~35 in the pLVX-IRES-Puro Group (negative control, NC). The expression of SV40LT in the pLVX-IRES-Puro-SV40LT group was significantly higher than the NC group (*p* < 0.01) (Figure 1B) confirming successful infection.

Lentiviruses were diluted at a multiplicity of infection (MOI) of 50 and 100 to infect primary RMCs (Pri RMCs). The infection efficiency at an MOI of 100 was higher than that of an MOI of 50 and was selected for subsequent experiments (Figure 1C). After puromycin screening, normal cultured Pri RMCs died, whilst some virus-infected cells survived. The remaining cells maintained the morphological characteristics of rabbit melanocytes. Cells were cultured into 96-well plates and four positive melanocyte cell lines were obtained (Figure 1D). A single cell line was selected for further passage and assessments.

The Im RMCs were cultured in primary melanocyte culture media and passaged in 0.25% trypsin. Cells were passaged at a ratio of 1:2.

### 2.2. Morphology and Proliferation of the Immortalized RMCs

**Figure 2:**
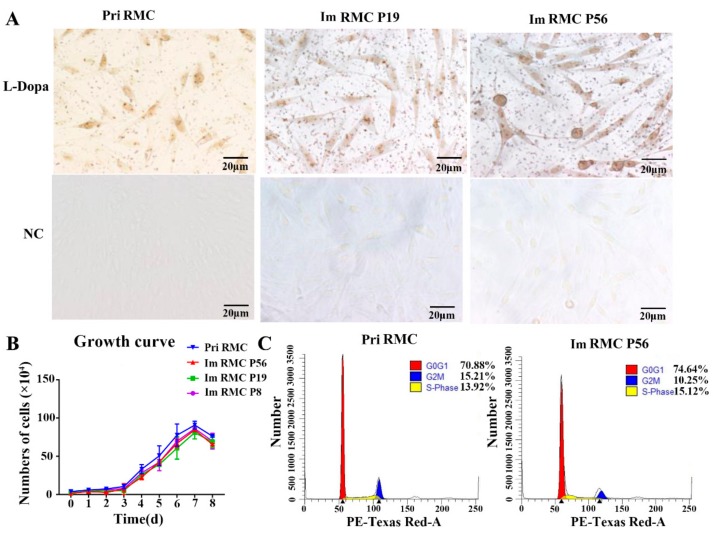
Morphology and proliferation of primary and immortalized melanocytes. (**A**) Identification of Dopa staining (400×). (**B**) Determination of growth curves. Im RMC P55/P19/P8 indicates the Im RMCs at passage 56/19/8. (**C**) Flow cytometry analysis. Pri RMC: primary RMC.

RMC P19 and RMC P56 cells were Dopa-positive. The morphology of the immortalized RMCs showed multipolarity which was comparable to primary cells (Figure 2A).

To assess cell proliferation, RMC P8, RMC P19 and RMC P56 cells were seeded into 24-well plates and counted every 24 h for 8 days. The immortalized RMCs had similar growth characteristics to primary cells, showing high levels of proliferative activity (*p* > 0.05) (Figure 2B).

The cell cycle status of primary melanocytes and RMC P56 cells were determined by flow cytometry. No significant differences in the number of cells in the S-phase between the cells were observed (*p* > 0.05), indicating that the immortalized RMCs maintained normal proliferation levels (Figure 2C).

### 2.3. Tissue-Specific Gene Expression in the Primary and Immortalized Melanocytes

**Figure 3:**
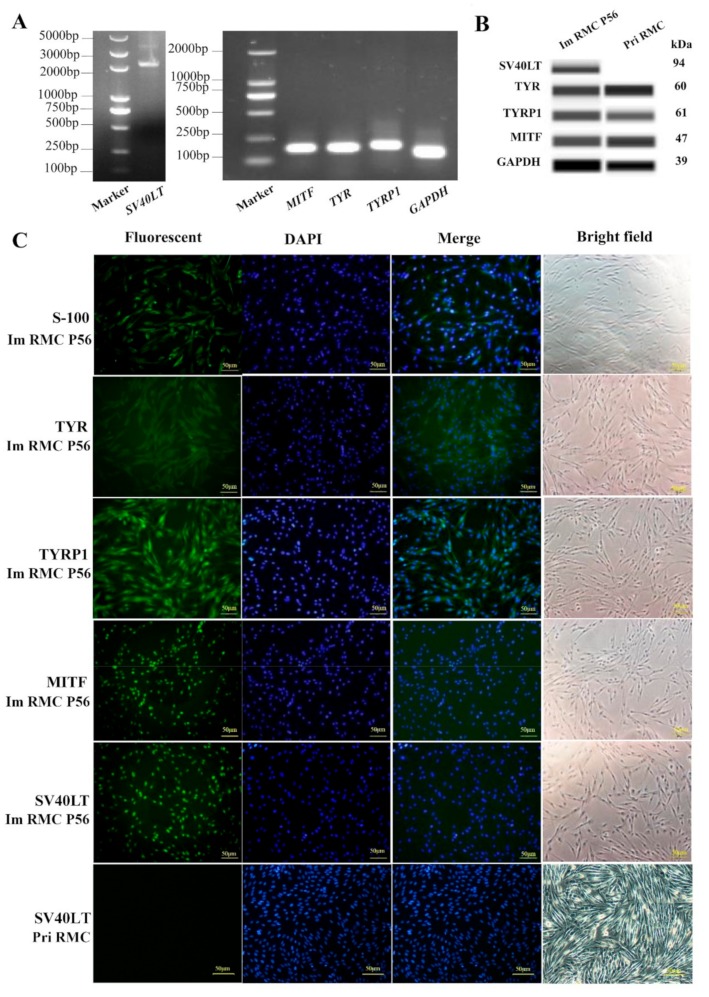
Tissue-specific gene expression in primary and immortalized melanocytes. (**A**) Expression of *SV40-LT*, *MITF*, *TYR*, *TYRP1,* and *GAPDH* in Im RMCs by SqPCR. (**B**) Western blot analysis and (**C**) immunofluorescence (200×) in Pri RMCs and Im RMCs.

RMC P56 cells were assessed for *SV40-LT*, *MITF*, *TYR*, *TYRP1*, and *GAPDH* mRNA expression by SqPCR (Figure 3A). *SV40-LT*-target band was observed in immortalized RMCs but was absent in Pri RMCs. Western blot and immunofluorescent staining showed SV40-LT expression in the RMCs, and no expression in the Pri RMCs, indicating successful integration (Figure 3B,C). Expression of the melanocyte-specific markers MITF, TYR, and TYRP1 were found in Im RMCs (Figure 3B,C). These findings were consistent with Pri RMC western blot (Figure 3B) and immunofluorescent analysis.

### 2.4. Malignant Transformation of Immortalized RMCs 

**Figure 4:**
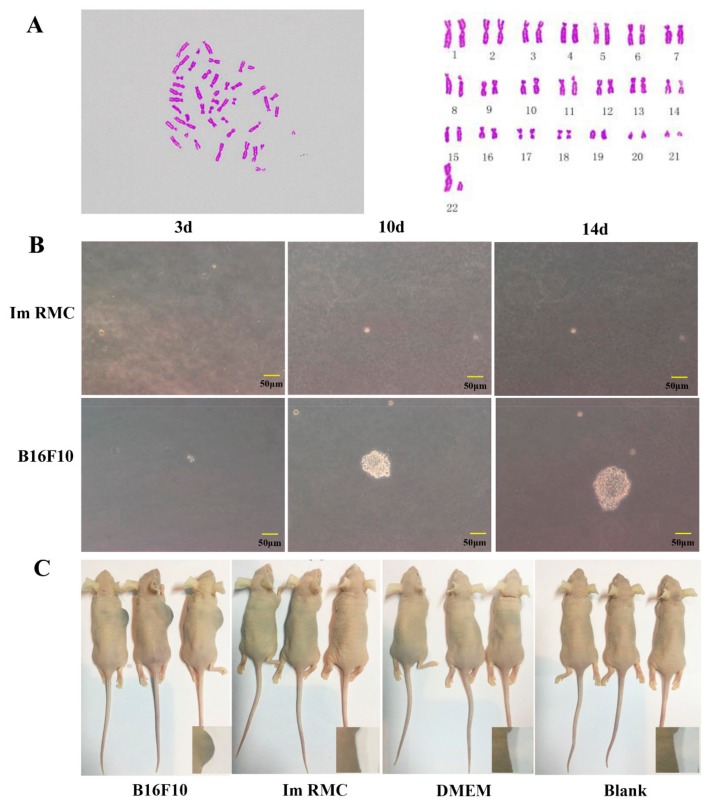
Malignant transformation of immortalized melanocytes. (**A**) Karyotype analysis of immortalized melanocytes (1000×). (**B**) Soft agar colony assays for the analysis of the malignant transformation of immortalized cells (200×). B16F10 was used as a positive control. (**C**) Tumor induction in nude mice. B16F10 was used as a positive control. Dulbecco’s Modified Eagle Medium (DMEM) medium was used as a negative control. Blank indicates no injection.

Karyotype analysis of the RMC P56 cells was performed, showing 44 chromosomes (22 pairs), consistent with normal rabbit chromosomes (Figure 4A). To detect malignant transformation in the immortalized RMCs, soft agar colony formation assays were performed. After RMCs were co-cultured in agar for 2 weeks, cell clones were visible in the mouse melanoma (B16F10) group, but absent in the RMC P56 group. These results indicated that the malignant transformation of immortalized RMCs did not occur despite SV40-LT integration (Figure 4B).

In nude mice injected with B16F10, RMC P56, and cell-free medium, the RMCs grew well but no tumor mass was formed. The nude mice injected with B16F10 developed black tumors at the inoculation site (Figure 4C). This confirmed that the immortalized RMCs had not undergone tumorigenesis.

## 3. Discussion

In general, primary cells proliferate to low levels in vitro. After culturing, primary cells typically develop vacuoles, which are accompanied by mitotic stagnation, aging, and death [19]. An array of methods to isolate and culture MCs in vitro have been described but these processes are both time-consuming and the lifecycle of the cells is limited. New immortalized MC lines are thus in highly demand. In this study, an immortalized rabbit melanocyte cell line was established, which maintained the morphological and proliferative characteristics of primary melanocytes, suggesting its utility as an in vitro study model for fur pigmentation studies.

Whilst primary cells are difficult to transfect, they are permissive to recombinant virus-mediated gene transduction. Lentiviral expression vectors stably introduced the target gene and enhanced its transcriptional activity [20]. We introduced SV40-LT into the primary RMCs using the lentivirus pLVX-IRES-Puro [21,22,23,24]. The primary cells were highly proliferative and healthy. We therefore selected a lentiviral system to package the pseudovirus and perform SV40-LT transductions.

To-date, many immortalized genes have been discovered, amongst which SV40 large T antigen is the most widely studied. SV40-LT is known to inactivate Rb through p53 to promote cell immortalization by preventing cell senescence and apoptosis [7,8,25]. In most cases, the T antigen enters the cell cycle by inducing the inactivation of Rb proteins (pRB, p130, and p107), thereby activating E2F-dependent transcription and promoting S phase progression, proliferation, and immortalization [26]. In addition, SV40-LT mediated cell immortalization can occur due to changes in telomerase activity and telomere fragmentation [26,27]. Thus, SV40-LT leads to the two-step immortalization of primary cells [28]. Through its interaction with p53 and pRB, SV40 T antigen abolishes their inhibition of the cell cycle and permits continuous proliferation. After multiple divisions, the cells are in a high-risk stage. During this period, most cells die and only a small number of cells activate intrinsic telomerase activity, thereby stabilizing telomeres, allowing the cells to grow indefinitely, obtaining immortality. A range of SV40-LT mediated immortalized cells have been constructed, which retain the differentiated phenotype of primary cells to reflect their biological characteristics [17,29,30,31]. According to those previous studies, we hypothesize that SV40-LT may interact with pRb and p53 or activate telomerase activity in Im RMCs, thereby achieving the immortalization of RMCs. The further analysis of mechanism of SV40-LT induced melanocytes immortalization will be our next step.

SV40-LT antigen induces chromosomal abnormalities and non-diploid cells, which increases genomic instability, promoting transformation [32]. However, the chromosome number and karyotype of Im RMCs remained normal, possibly due to the fact that the transduced SV40-LT gene did not cause chromosomal abnormalities, or that cells with chromosomal abnormalities at the mitotic checkpoint were removed. Soft agar assays showed that Im RMCs could not grow into clones in long-term soft agar culture, but B16F10 melanoma cells could form obvious clones. It is well known that B16F10 melanoma cells proliferate indefinitely and induce the overproduction of melanin that frequently leads to melanoma [33,34]. Karyotype analysis and soft agar growth assays showed that Im RMCs did not undergo tumorigenesis and acted normally.

In summary, the established melanocyte cell line proliferates indefinitely, but does not undergo tumorigenesis. This can overcome the issue of cell number, as can be used as a tool cell for future MC studies on the pigmentation mechanisms of fur animals.

## 4. Materials and Methods

### 4.1. Ethics Statement

This study was carried out in accordance with the recommendations of Animal Care and Use Committee at Yangzhou University. The experimental procedures was approved by the Animal Care and Use Committee at Yangzhou University (Yangzhou, China, 24 October 2017, No. 201710001). Operational procedures were stringently conducted in accordance with Laboratory Animal Requirements of Environment and Housing Facilities (GB14925-2001).

### 4.2. Isolation, Cultivation, and Passage of Primary RMCs

Primary rabbit skin melanocytes were separated by two-step enzymatic digestion from 1.5 × 1.5-cm^2^ sections of back skin from 2-month-old rabbits. White hair and subcutaneous fat tissue were removed and the tissues were digested in 0.25% Dispase II enzyme solution at 4 °C for 14–16 h. The epidermis and dermis were peeled off with fine sputum and digested in 0.25% trypsin at 37 °C for 8 min. Digested cells were cultured in M254-specific medium containing 10% fetal bovine serum (FBS, Gibco, Carlsbad, CA, USA). The medium was changed every 2 days to observe the growth state of the melanocytes. When cell growth reached ~90%, cells were digested with 1 mL of 0.25% trypsin for 1 min. Subsequently, cells were passaged at a ratio of 1:2 in 1% HMGS-2 (Human Melanocyte Growth Supplement-2, PMA-Free) (Gibco, Carlsbad, CA, USA) of M254 complete medium. Cells were maintained at 37 °C in a 5% CO_2_ incubator.

### 4.3. Immortalization of Primary RMCs Using Lentiviral Vectors

The vector pLVX-IRES-puro was purchased from Clontech (Clontech, CA, USA). The pLVX-IRES-Puro-SV40LT lentiviral expression vector was constructed and packaged. HEK293T cells were used for lentiviral packaging and viruses were collected for concentration. The infection efficiency of primary MCs was the highest at an MOI of 100. Cells were infected with lentiviruses for 72 h, and screened with 2.0 μg/mL puromycin for 1 week. Cells were maintained in puromycin (1.0 μg/mL) for 2 weeks.

### 4.4. Monoclonal Selection

Following puromycin selection, cells were counted so that 100 μL of medium contained 1 cell (1 cell per well of a 96-well plate). Monoclonal selections were performed on 96-well plates and cells were transferred to 48-well plates, 24-well plates, 12-well plates, 35-mm dishes, 60-mm dishes, and 25-cm^2^ culture flasks. Cells were cultured to more than 50 passages. SV40-LT expression was evaluated by immunofluorescence staining, semi-quantitative PCR and western blot analysis. Immortalized cell lines were maintained in M254 complete medium with 1% HMGS-2. 

### 4.5. Dopa Staining

Primary RMC P19 and RMC P56 cells were cultured in 24-well plates. When cell confluency reached 50–60%, cells were fixed at room temperature for 20–30 min. Melanocytes were treated with 0.1% L-Dopa (Sigma, Darmstadt, Germany) at 37 °C for 3 h, and replaced with fresh 0.1% L-Dopa at 37 °C for 3 h. After coloring, cells were rinsed three times in phosphate buffer saline (PBS), and imaged under an inverted microscope.

### 4.6. Immunofluorescence

RMC P56 cells were seeded into 24-well plates and fixed with 4% paraformaldehyde (Solarbio, Beijing, Tongzhou, China) for 20–30 min. Cells were permeabilized in 0.3% TritionX-100 (Solarbio, Beijing, Tongzhou, China) for 60 min and blocked in 1% bovine serum albumin (BSA, Boster, Wuhan, China), at for 60 min at RT. Cells were probed with antibodies to S-100 (1:500 mouse monoclonal, Boster, Wuhan, China), TYR (1:1000 rabbit polyclonal, BBI, Wuhan, China), TYRP1 (1:250 rabbit polyclonal, Abcam, Cambridge, UK), MITF (1:250 rabbit polyclonal, Santa Cruz, CA, USA), and SV40-LT (1:250 mouse monoclonal, Santa Cruz, CA, USA) and stained with goat anti-rabbit/mouse IgG H&L Fluorescein isothiocyanate (FITC) secondary antibodies (1:2500, Abcam, Cambridge, UK) at 37 °C for 60 min. Cells were DAPI (Boster, Wuhan, China) stained for 10 min and cells were imaged under a fluorescent inverted microscope.

### 4.7. Quantitative Real-Time PCR (qRT-PCR) and Semi-Quantitative PCR (Sq-PCR)

Primers for *SV40-LT* are shown in Table 1. GAPDH was used as an internal reference. Real-Time PCR was performed using ChamQTM SYBR® qPCR Msater Mix (Vazyme, Nanjing, China) on The Applied Biosystems® QuantStudio® 5 Real-Time PCR System with the following parameters: Pre-denaturation stage 95 °C for 30 s; PCR reaction 95 °C for 10 s, 60 °C for 30 s (40 cycles); dissolution at 95 °C for 15 s, 60 °C for 1 min, 95 °C for 15 s. The relative expression of the target gene was calculated using the △△Ct method = 2 (△Ct experimental − △Ct control) = 2^−△△Ct^. According to the relative quantification method, the expression of qRT-PCR was 1 in the control group.

### 4.8. Western Blot Analysis

Cells were lysed in radio-immunoprecipitation assay (RIPA, Sigma, Darmstadt, Germany) containing phenylmethanesulfonyl fluoride (PMSF, final concentration of 1 mM). Lysates were centrifuged at 10,000 rpm for 5 min at 4 °C. Supernatants were discarded and total proteins were obtained. Western blot analysis was performed using the Wes Simple Western system (Protein Simple, New York, USA). Proteins were transferred to membranes at 375 V for 25 min, blocked for 15 min, and probed with indicated primary antibodies for 30 min, followed by labeling with Anti-Mouse Secondary Antibody for 30 min (12–230 kDa Jess or Wes Separation Module, 8 × 25 capillary cartridges, Protein Simple, New York, USA). After 3 h, data were obtained analyzed using Compass software (Protein Simple, New York, USA). Primary antibodies included anti-TYR (1:100 rabbit polyclonal, BBI, Wuhan, China), anti-TYRP1 (1:100 rabbit polyclonal, Abcam, Cambridge, UK), anti-MITF (1:100 rabbit polyclonal, Santa Cruz, CA, USA), and anti-SV40-LT (1:100, mouse monoclonal, Santa Cruz, CA, USA).

### 4.9. Growth Curves

To evaluate the growth of established RMC cell lines, cells at passage 8, 19 and 56 were plated at a density of 2 × 10^4^ cells per well (24-well culture plate). Cells were counted using TC20TM Automated Cell Counters (BIO-RAD, California, USA) every 2 days through staining with trypan blue (Invitrogen & Clontech, Carlsbad, CA, USA). Growth curves were constructed and plotted as the culture time (d) against the cell number.

### 4.10. Cell Cycle Analysis

Primary melanocytes and Im RMC P56 were trypsinized and resuspended in chilled PBS. Cell Cycle and Apoptosis Analysis Kits (Beyotime, Shanghai, China) were used according to the manufacturer’s instructions. Cells were fixed in ice cold 70% ethanol for 12~24 h at 4 °C. We prepared propidium iodide (PI) staining solution, including 0.5 mL of staining buffer, 25 μL of PI staining solution (20×), and 10 μL of RNase A (50×) per sample. Cells were resuspended in 0.5 mL of PI at 37 °C for 30 min in the dark. Cell cycle analysis was performed on a Flow cytometer FACSAria SORP (Becton Dickinson) at 488 nm. DNA content analysis was performed using ModFit LT 5.0 software. The distribution of the cells at various cell cycle stages were obtained, and G0/G1%, S%, and G2/M% were calculated to assess proliferative capacity.

### 4.11. Karyotype Analysis

Chromosomes were prepared from RMCs at passage 56. Cells were exposed to 0.1 mg/mL colchicine (Invitrogen & Clontech, Carlsbad, CA, USA) in fresh medium and incubated at 37 °C. After 5–6 h, cells were trypsinized and collected by centrifugation at 1000 rpm for 10 min. Cells were treated with hypotonic buffer containing 0.075 mol/L potassium chloride (KCl) in a 37 °C water bath for 32 min. Cells were fixed and aspirated a pre-cooled slide. Slides were stained with Giemsa solution in PBS for 10 min at RT. 

### 4.12. Soft Agar Assays

B16F10 cell line was used as a positive control. B16F10 and Im RMC P56 (1 × 10^3^ cells/mL) were mixed with preheated 0.7% agarose and spread onto pre-packed 1.2% agarose plates. Experiments were performed in three replicate wells. Cells were cultured in a 5% CO_2_ incubator at 37 °C. Complete media was added every 2 days to prevent the agar from drying. Cells were imaged after 2-weeks of culture.

### 4.13. Tumorigenesis Assays

Im RMC P56 were collected and resuspended in serum-free medium. Cell suspensions (0.3 mL cell suspension, 1 × 10^6^ cells/mL) were subcutaneously injected into the right forelimb of 4-week-old female nude mice (Experimental Animal Center, Yangzhou University, China). Positive control groups were injected with 0.3 mL of B16F10 cell suspension (1 × 10^6^ cells/mL). Negative control groups were injected with 0.3 mL of serum-free DMEM medium. All experiments were performed on a minimum of three occasions.

### 4.14. Statistical Analysis

Each experiment was repeated on a minimum of three times. Statistical significance between experimental and control groups were analyzed by an independent sample test. The results are shown as the mean ± standard deviation (SD) at a significance level of **p < 0.01.

## Figures and Tables

**Table 1:** Primer sequences.

Name	Sequence(5′ to 3′)	Product Length/bp	Experiment
*SV40-LT*	*G***GAATTC** ATGGATAAAGTTTTAAACAG	2148	Cloning
	*CG***GAATTC** TTATGTTTCAGGTTCAGGG		
*hu GAPDH*	TGCACCACCAACTGCTTAGC	87	qRT-PCR
	GGCATGGACTGTGGTCATGAG		
*SV40-LT*	AAGTTTAATGTGGCTATGGG	92	qRT-PCR
	ACTGTGAATCAATGCCTGTT		
*ra MITF*	CCTCCAAGCCTCCGATAAGCTC	151	PCR
	TCACGGGCACTCTCTGTTGCAT		
*ra TYR*	GCACAACCGGGAATCCTACA	169	PCR
	CCAGATCCGACTGGCTTGTT		
*ra TYRP1*	AGCAATCCTGGGCTCAGTTC	190	PCR
	CCATCATGGGGGTAATGGGG		
*ra GAPDH*	CACCAGGGCTGCTTTTAACTCT	146	PCR
	CTTCCCGTTCTCAGCCTTGACC		

## Abbreviations

Pri RMC	primary rabbit melanocyte
Im RMC	immortalized rabbit melanocyte
Im RMC P56	immortalized rabbit melanocyte at passage 56
SV40-LT	simian virus 40 Large T
HPV	human papilloma virus
qRT-PCR	quantitative real time PCR
NC	negative control
Sq-PCR	semi-quantitative PCR
MOI	multiplicity of infection
DMEM	dulbecco’s Modified Eagle Medium
FBS	fetal bovine serum
HMGS-2	human melanocyte growth supplement-2
BSA	bovine serum albumin
RIPA	radio-immunoprecipitation assay
PMSF	phenylmethanesulfonyl fluoride
PBS	phosphate buffer saline
FITC	fluorescein isothiocyanate
KCL	potassium chloride
TYR	tyrosinase
TYRP1	tyrosinase related protein 1
MITF	microphthalmia-associated transcription factor
GAPDH	glyceraldehyde-3-phosphate dehydrogenase
